# Prevalence and associated factors of birth asphyxia among live births at Debre Tabor General Hospital, North Central Ethiopia

**DOI:** 10.1186/s12884-020-03348-2

**Published:** 2020-10-28

**Authors:** Wubet Alebachew Bayih, Getachew Yideg Yitbarek, Yared Asmare Aynalem, Biruk Beletew Abate, Aragaw Tesfaw, Metadel Yibeltal Ayalew, Demeke Mesfin Belay, Habtamu Shimelis Hailemeskel, Abebaw Yeshambel Alemu

**Affiliations:** 1 Debre Tabor University, Debra Tabor, Ethiopia; 2 Debre Berhan University, Debre Berhan, Ethiopia; 3 Woldia University, Weldiya, Ethiopia; 4 Bahir Dar University, Bahir Dar, Ethiopia

**Keywords:** Prevalence, Birth asphyxia, Ethiopia

## Abstract

**Background:**

More than one third of the neonatal deaths at Neonatal Intensive Care Unit (NICU) in Debre Tabor General Hospital (DTGH) are attributable to birth asphyxia. Most of these neonates are referred from the maternity ward in the hospital. Concerns have also been raised regarding delayed intrapartum decisions for emergency obstetrics action in the hospital. However, there has been no recent scientific evidence about the exact burden of birth asphyxia and its specific determinants among live births at maternity ward of DTGH. Moreover, the public health importance of delivery time and professional mix of labor attendants haven’t been addressed in the prior studies.

**Methods:**

Hospital based cross sectional study was conducted on a sample of 582 mother newborn dyads at maternity ward. Every other mother newborn dyad was included from December 2019 to March 2020. Pre-tested structured questionnaire and checklist were used for data collection. The collected data were processed and entered into Epidata version 4.2 and exported to Stata version 14. Binary logistic regressions were fitted and statistical significance was declared at p less than 0.05 with 95% CI.

**Results:**

The prevalence of birth asphyxia was 28.35% [95% CI: 26.51, 35.24%]. From the final model, fetal mal-presentation (AOR = 6.96: 3.16, 15.30), premature rupture of fetal membranes (AOR = 6.30, 95% CI: 2.45, 16.22), meconium stained amniotic fluid (AOR = 7.15: 3.07, 16.66), vacuum delivery (AOR =6.21: 2.62, 14.73), night time delivery (AOR = 6.01: 2.82, 12.79) and labor attendance by medical interns alone (AOR = 3.32:1.13, 9.78) were positively associated with birth asphyxia at 95% CI.

**Conclusions:**

The prevalence of birth asphyxia has remained a problem of public health importance in the study setting. Therefore, the existing efforts of emergency obstetric and newborn care should be strengthened to prevent birth asphyxia from the complications of fetal mal-presentation, premature rupture of fetal membranes, meconium stained amniotic fluid and vacuum delivery. Moreover, night time deliveries and professional mixes of labor and/delivery care providers should be given more due emphasis.

**Supplementary Information:**

**Supplementary information** accompanies this paper at 10.1186/s12884-020-03348-2.

## Introduction

Birth asphyxia or neonatal asphyxia or asphyxia neonatorum or perinatal asphyxia is defined as “failure to initiate and sustain spontaneous breathing at birth [1–4]. The parameter of Apgar score is used to determine the level of birth asphyxia, evaluated in the first and fifth minutes of life, with scores ranging from zero to ten [5]. A diagnosis of birth asphyxia can be made when a newborn has an Apgar score of < 7. Apgar score values of four to seven indicate moderate birth asphyxia whereas severe asphyxia is between zero and three [6]. Severe degrees of asphyxia can cause severe multiorgan damage resulting in brain **damage**, lung dysfunction, cardiomyopathy, renal failure, hepatic failure and necrotizing enterocolitis [7–27]. From these damages, brain damage is of the greatest concern because the survivors are likely to have lifetime complications like permanent seizure disorder, intellectual incompetence and motor deficits. This in turn has raised the demand for costly technological care of asphyxiated newborns though little can be done for severely asphyxiated neonates in the health care system [3–5].

Birth asphyxia can be caused by factors related to the antepartum, intra partum or post partum period [28–49]. However, quality of intrapartum care during labor and delivery has been recognized as the single most important predictor of the overall morbidity and mortality from asphyxia neonatorum [32, 34, 36–49]. More specifically, factors like antenatal obstetric complications [17, 20, 40, 42, 49–55], parity [19, 42], multiple births [22], gestational age < 37 or > 41 weeks [22, 42], low birth weight [18, 40–42], premature rupture of membranes [20, 44, 45, 48], prolonged labor [18, 19, 41, 42] and fetal distress [40, 41, 48, 49] have already been identified to be among the risk factors of birth asphyxia.

The Ethiopian neonatal mortality rate (NMR) is 30/1000 live births. The Amhara region, where the study was conducted, has the highest burden of the NMR, with 47/1000 live births [16]. Literature shows birth asphyxia is a universal public health problem with varied significance country wise [9, 15, 16]. For example, 31.6% of the Ethiopian neonatal mortality is attributed to birth asphyxia [24].

A significant number of neonatal deaths (33.3%) at Neonatal Intensive Care Unit (NICU) of Debre Tabor General Hospital (DTGH) are attributable to birth asphyxia. Most of these neonates are referred from the maternity ward in the hospital. Concerns have also been raised regarding delayed intrapartum decisions for emergency obstetrics action in the hospital [52, 56]. However, there has been no scientific evidence on the exact burden of birth asphyxia and its specific determinants among live births at maternity ward of DTGH. Moreover, public health importance of delivery time and professional mix of labor attendants are originally explored in the context of the study setting, which is helpful to make contextual interventions appropriate for optimizing feto-neonatal cardiopulmonary well being during pregnancy, labor and delivery.

## Methods

### Study setting, period and design

A hospital-based quantitative cross sectional study was conducted from December 2019 to March 2020 at maternity ward of Debre Tabor General Hospital. The hospital is located in Debre Tabor town 666 km from the capital of Ethiopia, Addis Ababa [56].

### Study participants

Mothers who gave live birth after 28 weeks of gestational age were screened for eligibility. Newborns of unknown gestational age at birth were excluded. Furthermore, newborns with malformations incompatible to life such as hydrops and cyanotic congenital heart defects were excluded as these newborns have already been predisposed for asphyxia.

### Sample size determination and sampling technique

A sample of 582 mother-newborn dyads was obtained by considering a confidence level of 95%, marginal error of 4%, *P* = 0.328 as of a prior study [17] and none response rate of 10%. The average monthly delivery rate of DTGH was 285 as of the last quarter report in 2018 [56]. Then, to ensure representativeness of the sample size, every other (k = 2) eligible mother newborn dyad was selected over 3 months at maternity ward of the hospital.

### Data collection procedure

An interviewer based questionnaire was used to collect primary data on maternal sociodemographic and ante partum related factors. For twin births, every mother was asked only once about socio-demographic and antenatal factors of her twin babies because twin neonates share similar socio-demography and antenatal history. Furthermore, a pretested structured checklist was employed to abstract secondary data from maternal chart on intra-partum (induction and/augmentation, fetal distress, fetal presentation at birth, mode of delivery, time of membrane rupture, duration of labor, color of amniotic fluid) and neonatal related factors (sex, birth weight, gestational age at birth and birth weight to gestational age). For twin births, every mother’s chart was reviewed twice for the aforementioned intra-partum and neonatal related characteristics due to variation of these factors between twin neonates.

Fifth minute APGAR score was directly collected by eight BSc graduating class midwifery students. The APGAR score was collected both during day and night shifts in the delivery ward and operation room. Apgar score was measured using its five components. Then, the score of each component was summed up and the information was documented using an Apgar score card (Supplementary file 1) [2, 3, 6, 53].

The diagnosis of birth asphyxia was made based on fifth minute APGAR score. Then, the diagnoses of birth asphyxia were further confirmed through medical interns’ consultation with obstetricians and pediatricians, and to determine the severity and management of the birth asphyxia.

The questionnaire (Supplementary file 2) and checklist (Supplementary file 3) were prepared after reviewing different articles conducted in Ethiopia [7, 16–18, 38–42, 53], other African [9, 19, 20, 45, 48, 49] and worldwide studies [1–3, 6, 8, 15, 21, 22, 27, 28, 35, 36, 46]. The questionnaire was validated through pretesting on 12 eligible mother- newborn dyads (5% of sample size) at DTGH 1 week before the actual data collection.

### Operational definitions

**Birth asphyxia:** A newborn was considered to have birth asphyxia when its fifth minute APGAR score was < 7 [2, 7, 53].

**Prolonged labor (second stage)**: For nulliparous mothers, if labor exceeds 3 h with provision of regional anesthesia, or 2 h without regional anesthesia. For multiparous mothers, if labor exceeds 2 h with regional anesthesia or 1 h in the absence of regional anesthesia. Prolonged labor was collected from the maternal chart [6, 7].

**Premature rupture of membrane** was diagnosed when fetal chorio-amnionic membranes rupture at any time before the onset of true labor [7].

### Statistical analysis

Data were coded, edited and double entered into epidata version 4.2. Then, *Stata* version 14 was used for analysis. Frequencies, proportion, summary statistics and cross tabulation were used. Variable candidacy (***P*** < 0.25) for multivariable analysis was determined after running bivariable analysis using binary logistic regressions. Then, multivariable logistic regressions were performed to investigate statistically significant predictors of birth asphyxia. Finally, statistical significance was declared at (***P*** < 0.05) using AOR with 95% CI.

### Ethics approval and consent to participate

For this study, the Ethics committee of Debre Tabor University approved the use of informed voluntary verbal consent. Then, the verbal consent was obtained from each of the respondents and documented by the data collectors in the space provided within the participant information sheet. A copy of the documented verbal consent was given to each respondent. Parental consent wasn’t required because all the interviewed mothers were 16 and above years of old.

## Results

### Socio -demographic factors

In this study, all 582 mothers approached agreed to participate, thus a response rate of 100%. More than half of the respondents, 316(54.3%) were rural residents. The majority of respondents, 89.0%) were married and more than one third of them (36.4%) were unable to read and write. The mean maternal age was 25.7 (SD = ± 5.86) years of whom 61.2% were between 20 and 34 years. One hundred and twenty-three mothers (21.1%) had history of adverse pregnancy outcome of which prematurity (42.3%) accounted for the highest proportion (Table 1).

**Table 1:** Socio-demographic factors of mothers who gave live birth at Debre Tabor General Hospital, 2020 (*n* = 582)

Factor	Response	Frequency	%
Residence	Rural	316	54.3
	Urban	266	45.7
Age (years)	16–19	55	9.5
	20–34	356	61.2
	> 34	171	29.4
Marital status	Married	518	89.0
	Widowed	34	5.8
	Separated	30	5.2
Religion	Orthodox	473	81.3
	Muslim	109	18.7
Occupation	House wife	181	31.1
	Governmental Employee	172	29.6
	Merchant	147	25.3
	Daily Labor	82	14.1
Educational Status	Unable to read and write	*212*	36.4
	No formal education but can read and write	98	16.8
	Primary education (1–8)	107	18.4
	Secondary education (9–12)	71	12.2
	College or University	94	16.2
Gravidity	< 3	263	45.2
	≥3	319	54.8
Parity	Primiparous	201	34.5
	Multiparous	381	65.5
Birth spacing (Years)	< 2	207	35.6
	≥2	375	64.4
History of adverse pregnancy outcome	Yes	123	21.1
	No	459	78.9
^a^If yes, which one?(*n* = 123)	Abortion	27	22.0
	Intrauterine fetal death	8	6.5
	Still birth	32	26.0
	Preterm	52	42.3
	Neonatal death	19	15.5

^a^multiple answers were given

### Ante partum related factors

Four hundred ninety five mothers [495(85.1%)] had attended antenatal care at public hospitals 296(59.7%) and public health center 199(40.3%). However, it was only about half of the mothers 254(51.3%) who had four and above antenatal care visits. About one tenth of the mothers 61(10.5%) ever used substance during their gestation of which ever alcohol users accounted for the most majority 48(78.7%). In the antenatal period, about one quarter, 135(23.2%) mothers were found to have obstetric complications. From these complications, preeclampsia/eclampsia accounted for the highest percentage 54 (40.0%) (Table 2).

**Table 2:** Factors related to the ante partum period among mothers who gave live birth at Debre Tabor General Hospital, 2020

Factor	Response	Frequency	*%*
ANC (*n* = 582)	Yes	495	85.1
	No	87	15.0
Number of ANC visits **(*n*** **= 495)**	< 4	241	48.7
	≥4	254	51.3
Obstetric complication during pregnancy (*n* = 582)	Yes	135	23.2
	No	447	76.8
^a^Type of complication **(*n*** **= 135)**	Preeclampsia/eclampsia	54	40.0
	Antepartum hemorrhage	36	26.7
	Anemia	61	45.2
	Infections	14	10.4
	Gestational diabetes	8	5.9
Ever used substance during pregnancy (*n* = 582)	Yes	61	10.5
	No	521	89.5
^a^Type of substance ever used during pregnancy **(*n*** **= 61)**	Alcohol	48	78.7
	Khat	25	41.0
	Cigarette	5	8.2

^a^multiple answers were given

### Intra partum related factors

Of the total respondent mothers, 412 (70.8%) had spontaneous labor onset. Majority of the mothers 484(83.2%) had intrapartum rupture of fetal membranes whereas premature rupture of membrane was reported among 98 (16.8%) mothers. Following membrane rupture, meconium stained amniotic fluid was observed among 149 (25.6%) mothers and similar report was obtained to the presence of night time delivery 150 (25.8%). At labor, about one fifth of the fetuses 111 (19.1%) were malpresented (Table 3). Among the mal-presentations, there were 65 (58.6%) breech presentations of which 42(37.8%) were delivered vaginally whereas the rest 23(20.7%) were delivered by cesarean section (Table 4).

**Table 3:** Factors related to the intra-partum period among mothers who gave live birth at Debre Tabor General Hospital, 2020 (*n* = 582)

Factor	Response	Frequency	%
Fetal presentation	Vertex	471	80.9
	Malpresentation	111	19.1
Labor type	Spontaneous	412	70.8
	Induced	100	17.2
	Augmented	70	12.0
Labor duration	Normal	362	62.2
	Prolonged	137	23.5
	Precipitated	83	14.3
Time of membrane rupture	PROM	98	16.8
	Intrapartum	484	83.2
Duration of ROM	Normal	534	91.8
	Prolonged	48	8.3
Color of amniotic fluid	Meconium stained	149	25.6
	Clear	433	74.4
Delivery time	Night	150	25.8
	Day	432	74.2
Mode of delivery	SVD	381	65.5
	Vacuum	90	15.5
	C/S	111	19.1
Labor attendant	Midwife with IESO	327	56.2
	Midwife with obstetricians	126	21.7
	Medical interns alone	129	22.2

**Table 4:** Cross tabulation of malpresentation versus mode of delivery among live births at Debre Tabor General Hospital (*n* = 111)

Malpresentation type	Mode of delivery		
	Vaginal	Cesarean	Total
Breech	42(37.8%)	23 (20.7%)	65 (58.6%)
Abnormal cephalic^a^	29 (26.1%)	17 (15.3%)	46 (41.4%)
Total	71 (63.9%)	40 (36.0%)	111 (100.0%)

^a^Face, brow or compound presentations

### Newborn related characteristics

More than half of the newborns, 303 (52.1%) were females. The mean gestational age at birth was 38.5 (±2.4) weeks and more than half of the newborns 325(55.8%) were term. Moreover, the mean birth weight was 2687.3 (±1481.3) grams and about one quarter of the newborns 144(24.7%) had low birth weight. At birth, there were 105(18.0%) newborns with health problems. About two third 69 (65.7%) of these problems was constituted by neonatal sepsis followed by birth injury 57(54.3%). There were 88(15.1%) twin newborns. Among the second twins, there were 16 (36.4%) neonates not in a cephalic presentation, all of which were delivered by vaginal breech extraction (Table 5).

**Table 5:** Newborn related characteristics at Debre Tabor General Hospital, 2020 (*n* = 582)

Factor	Response	frequency	%
Sex	Male	279	47.9
	Female	303	52.1
Birth outcome	Singleton	494	84.9
	Twin	88	15.1
Birth weight	< 2500	144	24.7
	≥2500	438	75.3
Gestational age at birth	Preterm	171	29.4
	Term	325	55.8
	Post term	86	14.8
Birth weight for Gestational age at birth	Appropriate for gestational age	458	78.7
	Small for gestational age	60	10.3
	Large for gestational age	64	10.0
^a^Neonatal medical problem at birth (*n* = 105)	Neonatal sepsis	69	65.7
	Birth injury	57	54.3
	Congenital malformation (compatible with life)	25	23.8
^a^Birth injury type (*n* = 57)	Gross caput succedaneum	24	42.1
	Cephalhematoma	31	54.4
	Subgaleal hemorrhage	19	33.3
	Fracture	5	8.8

^a^multiple responses were given

### Prevalence of birth asphyxia

**Fig. 1:**
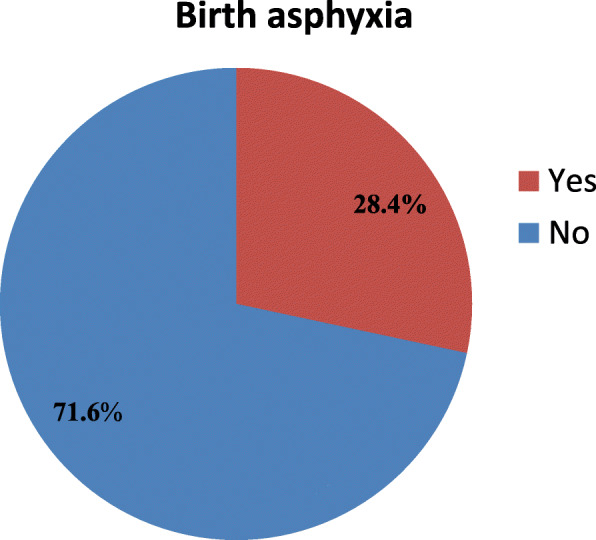
Prevalence of birth asphyxia among live births at DTGH, North Central Ethiopia, 2020 (*n* = 582)

**Fig. 2:**
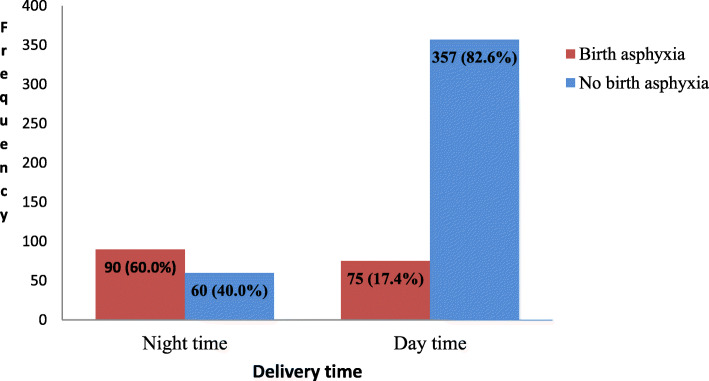
The prevalence of birth asphyxia by delivery time at DTGH, North Central Ethiopia, 2020 (*n* = 582)

**Fig. 3:**
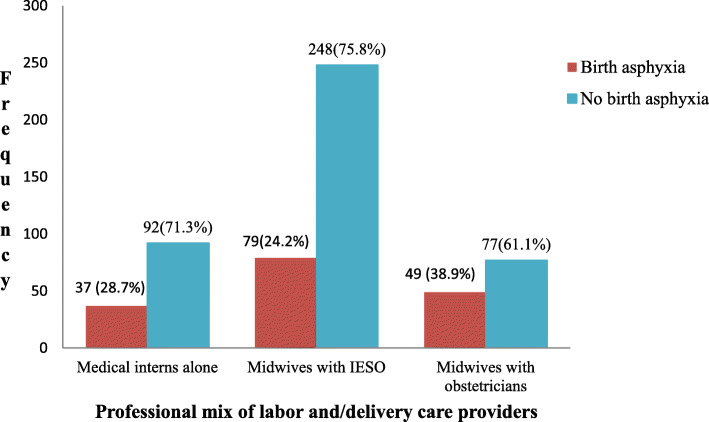
The prevalence of birth asphyxia by professional mix of labor and/delivery care providers at DTGH, North Central Ethiopia, 2020 (*n* = 582)

The prevalence of birth asphyxia was found to be 165 (28.3%) [95% CI: 26.5, 35.2%] (Fig. 1). Most of the asphyxiated neonates had moderate asphyxia 130 (78.8%) whereas 35 (21.2%) neonates had severe birth asphyxia. With respect to birth asphyxia among twin deliveries, the rate of birth asphyxia among the second twins (19.3%) was nearly twice higher than the rate among first twins (10.2%) (Table 6). The proportion of birth asphyxia among neonates delivered at night time 90(60.0%) was higher than those delivered at day time 75(17.4%) (Fig. 2). Moreover, the proportion of birth asphyxia among neonates delivered by the attendance of medical interns alone 37(28.7%) was higher than those delivered by the help of both midwives and obstetricians, 49(38.9%) (Fig. 3).

**Table 6:** Twin type versus birth asphyxia among live births at Debre Tabor General Hospital (*n* = 88)

Twin type	Birth asphyxia		
	Yes	No	Total
Twin A (first twin)	9 (10.2%)	35 (39.8%)	44 (50.0%)
Twin B (second twin)	17 (19.3%)	27 (30.7%)	44 (50.0%)
Total	26 (29.5%)	62 (70.5%)	88 (100.0%)

### Associated factors of birth asphyxia

The bivariable logistic regression analysis showed that 9 factors namely antenatal obstetric complications, fetal presentation, premature rupture of fetal membranes, meconium stained amniotic fluid, duration of labor, health professionals attending the labor and/delivery, mode of delivery, delivery time and birth outcome were crudely associated with birth asphyxia. However, after statistical adjustments in the final model, antenatal obstetric complications, duration of labor and birth outcome weren’t significant.

Neonates born with fetal mal-presentation had 7 times (AOR = 7.0, 95% CI: 3.2, 15.3) more likelihood of being asphyxiated at birth as compared to those of vertex presentations. Neonates born to mothers with premature rupture of fetal membranes were 6.3 times (AOR = 6.3, 95% CI: 2.5, 16.2) more prone to be asphyxiated at birth when compared to those with intrapartum rupture of membranes. Similarly, neonates born to mothers having meconium stained amniotic fluid were 7.2 times (AOR = 7.2, 95% CI: 3.1, 16.7) as likely to have birth asphyxia as compared to those born without being meconium stained. Regarding mode of delivery, neonates delivered by vacuum assisted vaginal route had 6.2 times (AOR =6.2, 95% CI: 2.6, 14.7) higher odds of association with birth asphyxia as compared to those born spontaneously. Furthermore, neonates delivered at night time had 6 times (AOR = 6.0, 95% CI: (2.8, 12.8) higher odds of association with birth asphyxia as compared to those delivered during the day time. Birth asphyxia was 3.3 times (AOR = 3.3, 95% CI: 1.1, 9.8) higher among neonates delivered by medical interns alone than those delivered by the attendance of midwives with IESO (Table 7).

**Table 7:** Bivariable and multivariable logistic regression analysis of factors associated with birth asphyxia among live births at DTGH, North Central Ethiopia, 2020 [*n* = 582]

Factor	Birth asphyxia		95% CI		*P* value
	Asphyxiated (*n* = 165)	Not asphyxiated (*n* = 417)	COR AOR(95%CI)	AOR	
**Antenatal obstetric complications**					
Yes	100	35	16.8 (10.5, 26.8)	6.5 (0.6, 1.6)	.08
No	65	382	1.0	1.0	
**Fetal presentation**					
Vertex	81	390	1.0	1.0	
Malpresentation	84	27	15.0 (9.1, 24.6)	7.0 (3.2, 15.3)	.000
**Membrane rupture**					
PROM	72	26	3.2 (1.7, 6.1)	6.3 (2.5, 16.2)	.000
Intra-partum	93	391	1.0	1.0	
**Amniotic fluid**					
Meconium stained	102	47	12.8 (8.2, 19.7)	7.2 (3.1, 16.7)	.000
Not stained	63	370	1.0	1.0	
**Delivery time**					
Night	90	60	7.1 (4.7, 10.8)	6.0 (2.8, 12.8)	.000
Day	75	357	1.0	1.0	
**Labor duration**					
Normal	50	312	1.0	1.0	
Prolonged	70	67	6.5 (4.2, 10.2)	5.5 (0.4, 1.6)	.21
Precipitated	45	38	7.4 (4.4, 12.5)	2.8 (0.1, 7.2)	.098
**Mode of delivery**					
SVD	58	323	1.0	1.0	
Vacuum	37	53	3.9 (2.4, 6.4)	6.2 (2.6, 14.7)	.001
CS	70	41	9.5 (5.9, 15.3)	6.5 (0.7, 2.0)	.065
**Labor attendants**					
Midwife with IESO	79	248	1.0	1.0	
Midwife with obstetricians	49	77	2.0 (1.3, 3.1)	1.4 (0.5, 3.4)	.517
Medical interns alone	37	92	1.3 (0.8, 2.0)	3.3 (1.1, 9.8)	.029
**Birth outcome**					
Singleton	139	355	1.0	1.0	
Twin	26	62	1.1 (0.7, 1.8)	1.2 (0.4, 3.3)	.717

## Discussion

In this study, the burden and predictors of birth asphyxia among live births at Debre Tabor General Hospital are reported. Fetal mal-presentation, premature rupture of fetal membranes, meconium stained amniotic fluid, vacuum assisted delivery, night time delivery and labor attendance by medical interns alone were found to be significant predictors of birth asphyxia.

From the study, the prevalence of birth asphyxia (28.4%) is consistent with a study at Dilla Referral Hospital in Southern Ethiopia (32.8%) [17]. However, the prevalence in our study was lower than the prevalence in Jimma zone public hospitals, South West Ethiopia (47.5%) [18] which could be attributed to the fact that our study was conducted at a single general hospital which serves a relatively less complicated referral deliveries than the presence of specialized hospital in Jimma zone public hospitals where more complicated referral deliveries are anticipated including birth asphyxia. Furthermore, the prevalence of birth asphyxia in our setting was lower than a study in Iran (58.8%) [21] which may be due to differences in sample size and study setting. Most importantly, difference in case definition might have played contribution for the variation; for example, our study was based on only fifth minute APGAR score less than 7 whereas that of the Iranian study used to have a flexible diagnostic criteria of birth asphyxia including: umbilical cord pH < 7 or 5 min Apgar score < 6 or 20 min Apgar score less than 7 or multi organs failure in the first 72 h or convulsion in the first 24 h of life.

In the analysis of associated factors, neonates born with fetal mal-presentation were 4.5 times more likely of to be asphyxiated as compared to those with vertex presentations. This finding was congruent with other Ethiopian studies [18, 40] where the odds of birth asphyxia among the mal-presented fetuses were 7 times and 4.5 times higher as compared to the vertex presented fetuses respectively. A Cameroonian study [20] also showed similar finding. This similarity could be due to the fact that mal-presentation is often associated with premature rupture of membrane, a factor of significance in this study. Following premature membrane rupture, newborn life threatening events like umbilical cord accidents (cord prolapse and cord compression) occur with subsequent asphyxia at birth [6]. Besides, though no statistical significance in this study, mal-presentation is often followed by prolonged labor which is a known obstetric emergency endangering feto-neonatal life [2, 6, 53]. Therefore, mothers should know their fetal presentation in the late gestation and those with fetal mal-presentation during labor should be given strict partographic follow up of fetal heart beat to early detect any fetal derangement for immediate action like operative deliveries [6, 7].

Neonates born to mothers with premature rupture of fetal membranes (PROM) were 6.3 times more prone to be asphyxiated at birth as compared to those with intrapartum rupture. A consistent finding was obtained from studies at Cameroon [20], Uganda [45] and Al-Diwaniya teaching hospital [48]. The consistence can be justified by the fact that when fetal membranes rupture prematurely, spontaneous gush of amniotic fluid happens along with the prolapse of umbilical cord which is in turn accompanied with cord compression and subsequent asphyxia at birth. Moreover, premature rupture of membranes, if prolonged, often facilitates feto-maternal systemic infections (3, 6, 7) which is usually ensued by subsequent neonatal asphyxia. Hence, mothers with PROM should be provided with emergency obstetric care. Besides, to prevent feto-neonatal infection, mothers with PROM should be given prophylactic antibiotics. Digital vaginal examinations should also be kept as minimal as possible to reduce the likelihood of ascending genital infections [6, 7, 24].

Pertaining to mode of delivery, vacuum delivery had 6.2 times higher odds of association with birth asphyxia as compared to the spontaneous vaginal deliveries. This could be due to the fact that vacuum delivery may result in hemorrhagic cranial injuries such as cephalhematoma and subgaleal hemorrhage, which could in turn be accompanied with birth asphyxia from the hemorrhagic anemia [6]. Moreover, painful nature of the vacuum procedure is claimed to depress the neonatal respiratory center in the brain stem [2, 6]. Hence, if vacuum assisted delivery is indicated, it should be maneuvered according to the strict procedure to cause as little pain and cranial injury as possible.

The risk of birth asphyxia among neonates with history of meconium stained amniotic fluid was 7.2 times higher than those born to mothers with clear amniotic fluid. This finding was consistent with other studies [17, 18, 40–42, 45–47]. The likely justification could be due to the fact that meconium stained amniotic fluid may be ensued by intrapartum inhalation of the meconium in the fluid which leads to mechanical obstruction of airways, surfactant inactivation, chemical inflammation and apoptosis of the pulmonary tissues thereby facilitating pulmonary air leak and hypoxia [6, 54]. Hence, meconium stained amniotic fluid should always be considered as a signal of birth asphyxia so that delivery care providers should be ready to give immediate resuscitation for such neonates [6, 24].

Night time delivery was a factor of statistical significance as shown by its 6 times higher odds of association with birth asphyxia when compared to day time delivery. This may be due to the fact that as compared to the day time, relatively fewer labor attendants are assigned to attend their respective duty hours over the burdensome night time. Furthermore, delayed arrival of senior obstetricians and IESO when consulted for difficult labors might have played roles [52, 56]. The higher odds of birth asphyxia during the night time may also be related to other unmeasured factors that could be the topic of a separate study.

Neonates that were delivered by the attendance of medical interns alone had 3.3 times higher odds of association with birth asphyxia than those delivered by the help of midwives and IESO. This could be due to studentship of medical interns who may not have the required experience of attending labor and delivery [56]. Thus, every labor should be attended by a professional mix of midwives, IESO and obstetricians to optimize intrapartum care, thereby enabling the mitigation of birth asphyxia.

### Limitation of the study

Despite the robust methodology we employed and the aforementioned findings we reached, our results are limited to the context of the study setting (Debre Tabor General Hospital). Moreover, to make diagnosis of birth asphyxia, only fifth minute APGAR score was considered due to lack of facilities for umbilical cord blood Ph and arterial blood gas analyses in the study setting. Quality of the intra partum obstetric and newborn care and maternal satisfaction towards the care weren’t addressed. The study also shares drawbacks of a cross-sectional design because the authors couldn’t establish causal association between the considered factors and occurrence of birth asphyxia.

## Conclusions

In the study area, the burden of birth asphyxia has remained a problem of public health importance. To the specific context of the study setting, night time delivery and attendance of labor by medical interns alone were positively associated with birth asphyxia. Furthermore, similar to various prior studies, fetal mal-presentation, premature rupture of fetal membranes, meconium stained amniotic fluid and vacuum delivery were significantly associated with increased odds of birth asphyxia. Most of these factors can be prevented even with our limited resources as recommended below.

## Recommendation

The burden of birth asphyxia in the study area can be mitigated through accountable performance of the following stakeholders in charge of optimizing neonatal health.

### Maternal and neonatal health care providers

Maternal and neonatal health care providers should make exhaustive investment of their efforts for early detection of antenatal and intra-partum emergency obstetrics through strict feto-maternal follow ups aided by different diagnostics (Eg. ultrasound). Besides, their early detection of abnormality should always be accompanied with immediate decisions for emergency obstetrics and newborn care interventions.

### Debre Tabor general hospital

The hospital should have to have an accountably scheduled professional mix of labor and delivery care providers (medical interns, midwives, IESOs and obstetricians) for every laboring mother. Moreover, the hospital management should perform duty supervision sessions at night time to identify the likely barriers of optimizing neonatal health at birth. Daily morning time sessions of delivery summary report and further discussions should be conducted to ensure a reasonable accountability of birth attendance in the hospital. The labor and delivery care providers should be supervised to determine whether they have the required skill of maneuvering vacuum assisted vaginal delivery, which in turn helps arrange the need for training of vacuum delivery.

### Debre Tabor University (DTU) and researchers

DTU, as an academic institution providing community service to the study hospital catchment, should conduct qualitative studies to determine quality of the intrapartum care. Besides, exploring maternal and delivery care providers’ reasons, feelings and experience of the challenges for optimizing birth outcome would have of public health importance to design feasible quality improvement projects at maternity ward in the hospital.

## Supplementary Information

**Additional file 1: Supplementary file 1.** Apgar score card: An Apgar score card used for determining the fifth minute APGAR score of every selected neonate at DTGH, North Central Ethiopia, 2020 [*n* = 582].

**Additional file 2: Supplementary file 2.** Questionnaire: A structured questionnaire used for interviewing selected mothers about their socio-demographic and antenatal characteristics, DTGH, North Central Ethiopia, 2020 [*n* = 582].

**Additional file 3: Supplementary file 3.** Checklist: A structured checklist used for abstracting intra-partum and neonatal related characteristics from the delivery summary of maternal chart at DTGH, North Central Ethiopia, 2020 [*n* = 582].

## Data Availability

Data will be available upon request from the corresponding author.

## Abbreviations

WHO: World Health Organization
ANC: Ante Natal Care
APGAR: Appearance, Pulse Rate, Grimace, Activity, Respiration Rate
CS: Cesarean Section
DTGH: Debre Tabor General Hospital
EDHS: Ethiopian Demographic Health Survey
FMOH: Federal Ministry of Health
GA: Gestational Age
IESO: Integrated Emergency Surgery Obstetrics officers
MSAF: Meconium Stained Amniotic Fluid
NICU: Neonatal Intensive Care Unit
NMR: Neonatal Mortality Rate
PROM: Premature Rupture of Membrane
SVD: Spontaneous Vaginal Delivery
