# Supplementary material for: Prevalence and associated factors of birth asphyxia among live births at Debre Tabor General Hospital, North Central Ethiopia
Source: BMC Pregnancy Childbirth. 2020 Oct 28;20:653. doi: 10.1186/s12884-020-03348-2 (PMC7594464; doi:10.1186/s12884-020-03348-2)
Supplement: Supplementary file 1 — Additional file 1: Supplementary file 1. Apgar score card: An Apgar score card used for determining the fifth minute APGAR score of every selected neonate at DTGH, North Central Ethiopia, 2020 [n = 582]. [file 12884_2020_3348_MOESM1_ESM.docx]

**APGAR score card**

**Registration code ____________________**

| **Component** | **Score 0** | **Score 1** | **Score 2** |
| --- | --- | --- | --- |
| **Activity** | Flaccid | Some flexion of limbs | Well flexed |
| **Pulse rate** | Absent | <100 per minute | >100 per minute |
| **Grimace** | No response | Grimace | Cough or sneeze |
| **Appearance** | Pale/Blue body | Blue extremities | Completely pink body |
| **Respiration** | Absent/gasping | Weak | Good cry |

***Total neonatal APGAR score out of 10* = _____**
