# Supplementary material for: Prevalence and associated factors of birth asphyxia among live births at Debre Tabor General Hospital, North Central Ethiopia
Source: BMC Pregnancy Childbirth. 2020 Oct 28;20:653. doi: 10.1186/s12884-020-03348-2 (PMC7594464; doi:10.1186/s12884-020-03348-2)
Supplement: Supplementary file 2 — Additional file 2: Supplementary file 2. Questionnaire: A structured questionnaire used for interviewing selected mothers about their socio-demographic and antenatal characteristics, DTGH, North Central Ethiopia, 2020 [n = 582]. [file 12884_2020_3348_MOESM2_ESM.docx]

**Debre Tabor University**

**College of Health sciences**

**Department of Pediatrics and Neonatal Health Nursing**

How are you? My name is __________________. I am working as a data collector for the study being conducted in this institution on the assessment of Prevalence of Birth Asphyxia and Associated Factors among Live Births at Debre Tabor General Hospital, North Central Ethiopia: 2020 proposed by Wubet Alebachew, who is maternity and neonatal nursing lecturer at Debre Tabor University. Findings of this study will be used as evidence to plan appropriate antenatal and intrapartum interventions to optimize newborn survival. Therefore, though you are autonomous in deciding your participation in the study, I kindly request you to be participant provided that you can withdraw at any time if you aren’t comfortable with. The information which you will provide is kept confidentially. Moreover, no particular reference will be made in oral or written reports that link you to the research.

Name and signature of the participant __________________date___________

Signature of data collector___________________________ date__________

***This voluntary consent form will be signed face to face in the presence of the data collector and its copy should be given to the participant!!!!!!***

**Thank you for your cooperation!!**

**Questionnaire code ………**

**General instruction**

**1.** For multiple choice questions, choose the best answer

**2**. If your answer is not listed among alternatives, please tell your own answer for the data collector.

Part I: Socio -demographic factors

| S/N | Factors | Response |
| --- | --- | --- |
| 100 | Residence | 1. Rural |
|  |  | 1. Urban |
| 101 | Age (years) | ________________ |
| 102 | Marital status | 1. Married 2. Widowed 3. Separated |
| 103 | Religion | 1. Orthodox 2. Muslim 3. Protestant |
| 104 | Occupation | 1. House wife 2. Governmental Employee 3. Merchant 4. Daily Labor |
| 105 | Educational Status | 1. Unable to read and write 2. No formal education but can read and write 3. Primary education (1-8) 4. Secondary education (9-12) 5. College or University |
| 106 | Gravidity | ____ |
| 107 | Parity | ____ |
| 108 | Birth spacing (Years) | ____ |
| 109 | History of adverse pregnancy outcome? | 1. Yes |
|  |  | 1. No |
| 110 | ^*^If yes, which one? | A. Abortion  B. Intrauterine fetal death  C. Still birth  D. Preterm  E. Neonatal death  F. Other |

^*multiple answers are possible^

Part II: Ante partum related factors

| S/N | Factor | Response |
| --- | --- | --- |
| 200 | ANC | 1. Yes 2. No |
| 201 | Number of ANC visits | ________ |
| 202 | Obstetric complication during pregnancy | 1. Yes 2. No |
| 203 | ^*^Type of complication | A. Preeclampsia/eclampsia  B. Antepartum hemorrhage  C. Anemia  D. Infections  E. Gestational diabetes  F. Other |
| 204 | Ever used substance during pregnancy | 1. Yes 2. No |
| 205 | ^*^ T Type of substance ever used during pregnancy | A. Alcohol  B. Khat  C. Cigarette  D. Other |

^*multiple answers are possible^
