# Supplementary material for: Prevalence and associated factors of birth asphyxia among live births at Debre Tabor General Hospital, North Central Ethiopia
Source: BMC Pregnancy Childbirth. 2020 Oct 28;20:653. doi: 10.1186/s12884-020-03348-2 (PMC7594464; doi:10.1186/s12884-020-03348-2)
Supplement: Supplementary file 3 — Additional file 3: Supplementary file 3. Checklist: A structured checklist used for abstracting intra-partum and neonatal related characteristics from the delivery summary of maternal chart at DTGH, North Central Ethiopia, 2020 [n = 582]. [file 12884_2020_3348_MOESM3_ESM.docx]

Structured checklist to abstract intra-partum and neonatal related factors

1. Intra-partum factors

| S/N | Factor | **Response** |
| --- | --- | --- |
| 300 | Presentation | 1. vertex 2. Breech 3. Face 4. Brow |
| 301 | Labor type | 1. Spontaneous 2. Induced 3. Augmented |
| 302 | Labor duration | 1. Normal 2. Prolonged 3. Precipitated |
| 303 | Time of membrane rupture | 1. PROM 2. Intrapartum |
| 304 | Duration of ROM (hours) | _____ |
| 305 | Color of amniotic fluid | 1. Meconium stained 2. Clear |
| 306 | Delivery time | 1. Night 2. Day |
| 307 | Mode of delivery | 1. SVD 2. Vacuum 3. C/S |
| 308 | Labor attendant | 1. Midwife with IESO 2. Midwife with obstetricians 3. Medical interns alone |

1. Newborn related factors

| S/N | Factor | Response |
| --- | --- | --- |
| 400 | Sex | 1. Male 2. Female |
| 401 | Birth outcome | 1. Singleton 2. Twin |
| 402 | Birth weight (grams) | _____ |
| 403 | Gestational age at birth (weeks) | _____ |
| 404 | Birth weight for Gestational age at birth | 1. Appropriate for gestational age 2. Large for gestational age 3. Small for gestational age |
| 405 | Admission diagnosis | 1. Neonatal sepsis 2. Neonatal jaundice 3. Congenital malformation (compatible with life) 4. Birth injury (state the type) __ 5. Other |

^*multiple answers are possible^
